# Being Conscious of Water Intake Positively Associated with Sufficient Non-Alcohol Drink Intake Regardless of Seasons and Reasons in Healthy Japanese; the KOBE Study: A Cross Sectional Study

**DOI:** 10.3390/ijerph16214151

**Published:** 2019-10-28

**Authors:** Tomofumi Nishikawa, Naomi Miyamatsu, Aya Higashiyama, Yoshimi Kubota, Yoko Nishida, Takumi Hirata, Daisuke Sugiyama, Kazuyo Kuwabara, Sachimi Kubo, Yoshihiro Miyamoto, Tomonori Okamura

**Affiliations:** 1 Foundation for Biomedical Research and Innovation, Hyogo 650-0047, Japan; miyan@belle.shiga-med.ac.jp (N.M.); ahigashi@ncvc.go.jp (A.H.); yo-kubota@hyo-med.ac.jp (Y.K.); y_nishida@fbri.org (Y.N.); tkm2006diabetes@yahoo.co.jp (T.H.); dsugiyama@z8.keio.jp (D.S.); kuwabara@a6.keio.jp (K.K.); kubo@fbri.org (S.K.); miyamoty@ncvc.go.jp (Y.M.); okamura@z6.keio.jp (T.O.); 2 Faculty of Health Science, Kyoto Koka Women’s University, Kyoto 615-0822, Japan; 3 Department of Clinical Nursing, Shiga University of Medical Science, Shiga 520-2192, Japan; 4 Department of Preventive Medicine and Epidemiologic Informatics, National Cerebral and Cardiovascular Center, Osaka 565-8565, Japan; 5 Department of Environmental and Preventive Medicine, Hyogo College of Medicine, Hyogo 663-8501, Japan; 6 Department of Preventive Medicine and Epidemiology, Tohoku Medical Megabank Organization, Tohoku University, Sendai 980-8573, Japan; 7 Department of Preventive Medicine and Public Health, School of Medicine, Keio University, Tokyo 160-8582, Japan

**Keywords:** water intake conscious, non-alcohol drink, seasons, daily salt intake, serum osmolarity, cross-sectional study

## Abstract

The present study sought to clarify if being conscious of water intake (CWI) is associated with sufficient non-alcohol drink (NAD) intake. We used data of healthy participants without diabetes, aged 40–74 years, in the Kobe Orthopedic and Biomedical Epidemiologic (KOBE) study. The association between being CWI and NAD intake was evaluated by multivariate linear regression analyses after adjusting for age, sex, surveyed months (seasons), alcohol drinking, health-awareness life habits, socioeconomic factors, serum osmolarity, estimated daily salt intake, and reasons for NAD intake. Among 988 (698 women and 290 men) participants eligible for the present analyses, 644 participants (65.2%) were CWI and 344 participants (34.8%) were not CWI (non-CWI). The most popular reason for being CWI was to avoid heat stroke in summer and to prevent ischemic cerebral stroke in winter. The CWI group took more NAD, especially decaffeinated beverages, than the non-CWI group (1846.7 ± 675.1 mL/day vs. 1478.0 ± 636.3 ml/day, *p* < 0.001). There was a significant association between being CWI and NAD intake in multivariate linear regression analyses ever after adjusting for the relevant variables (β = 318.1, *p* < 0.001). These findings demonstrated CWI, regardless of the reasons and the seasons, was associated with high NAD intake in Japanese healthy population.

## 1. Introduction

In contrast to most developed countries, the event risks of cerebrovascular diseases are high and it remains one of top-ranked causes of death and disability in Japan [1,2]. Recent studies demonstrated that patients with cerebral infarction (CI) took less water before the onset than healthy subjects [3], and higher water intake reduced mortality due to ischemic stroke [4,5,6,7,8]. The potential association between water intake and CI is generally explained via the following pathophysiological mechanism; 1) insufficient hydration is one of the most important factors causing dehydration and 2) dehydration is considered to be associated with the development of cerebral ischemic events [7,9,10], probably because of vascular collapse [11], increased blood viscosity [12], and spasm [13].

Water intake is predominantly through consumption of drinking water and beverages (80%) [14]. Therefore, non-alcohol drink (NAD) intake is commonly recommended for prevention of heat stroke in hot seasons because dehydration, which is one of the important causes of heat stroke, is commonly believed to occur in summer. However, disruption of hydration status does not necessarily occur in hot summer [15] and dehydration in winter should not be neglected because the body’s thirst response is decreased in cold temperatures [16] and cold-induced diuresis [17]. Accordingly, the occurrence of ischemic stroke has been reported to rise with decreasing temperature [18] and its incidence does not vary significantly with season [19,20]. Moreover, we also demonstrated a positive association between a lesser NAD intake habit and CI after adjusting for the effect of the seasonal change [3]. These findings strongly indicated maintaining sufficient water intake for prevention of CI due to dehydration not only in summer but also throughout the year is important. 

In order to maintain sufficient water intake throughout the year, keeping conscious of water intake (CWI) regardless of seasons is important because special attention for adequate supply of fluid is thought to be required [21]. However, the association between CWI and water intake has not been reported [14]. The present study therefore aimed to clarify the association between being CWI and NAD intake, taking seasons into consideration.

## 2. Materials and Methods 

### 2.1. Study Population

We performed a cross-sectional study of participants in the Kobe Orthopedic and Biomedical Epidemiological study (KOBE study), which is a population-based prospective cohort study of risk factors for cardiovascular disease or worsening of quality of life in Kobe, one of the major urban areas in Japan. The study participants were volunteers aged 40 to 74 years who were residents of Kobe; the participants had to meet the following criteria: 1) Not currently on medications for hypertension, dyslipidemia, and diabetes mellitus; and 2) no past history of cardiovascular diseases and cancer. The KOBE study started in 2010, and 1117 subjects (776 women and 341 men) participated in the baseline survey from July 2010 to December 2011, and they were followed up on every two years after the baseline survey; details have been previously reported [22,23,24,25,26]. The first follow-up survey was conducted between September 2012 and May 2014, in which the participants were asked whether they were CWI or not. Among 1117 subjects who participated in the first follow-up survey, 76 did not express their will to participate in the first follow-up survey, 8 stopped the participation, 8 died between the baseline survey and the first follow-up survey, 15 had missing information (11 did not attend the face-to-face interviews as well as on-site tests, 4 did not complete the blood test) and 1 did not take a urine test. In these 1009 participants, those who were diagnosed with diabetes on the basis of fasting glucose of ≥ 7.0mmol/L (n = 10) and/or HbA1c level of ≥ 6.5% (n = 19) in the follow-up survey (8 participants were included in the both diabetes criteria) were excluded; thus the data of 988 (698 women and 290 men) were used for the following analyses. These participants did not have a history of stroke. Written informed consent was obtained from all participants. The present study was approved by the Ethics Committee of the Institute of Biomedical Research and Innovation (Committee approval number: 11–12). 

### 2.2. Evaluation of Non-Alcoholic and Alcoholic Drink Intake

The research doctor, nurse, or nutritionist performed face-to-face interviews with each participant and reviewed the answers to the questionnaires that were distributed by post before the first follow-up survey. In reference to a study on the association between water intake and coronary heart diseases [27], questionnaires for NAD intake assessed the type of beverages as well as their cup sizes for each drink and the frequency to calculate the average volume of NAD intake per day (mL/day) in the season the survey was conducted. During the interview, one plastic bottle (500 ml) and three kinds of cups in different sizes, i.e., 300 ml, 200 ml, and 150 ml, were used to assist the interview process. Alcohol beverages and water in meals or soup were not included in the consumption of NAD. 

We also assessed alcoholic drink intake as described elsewhere [28]; briefly, the frequency of alcohol consumption during a recent typical week and total alcohol intake on each occasion were determined and used to calculate the ethanol intake per week. This value was then divided by 7 to obtain the mean ethanol intake per day. 

### 2.3. Data Collection for Characteristics

Data collection for characteristics at the follow-up survey was conducted in accordance with the baseline survey, which has been detailed in our previous studies [22,23,24,25]. Each subject completed a self-reported questionnaire to assess past medical history and life habits, including being CWI or not, reasons for being CWI, current smoker, current drinker, so-called health conscious life habits in the questionnaire (unhealthy life habits: Eating quickly, dinner within 2 hours before going to bed, late evening snack, skipping breakfast, and eating until full stomach; healthy: Regular use of vitamin tablets or supplements, walking habit, and daily use of a pedometer; the others: Sleeping time and self-rated health), and socioeconomic factors (living alone, educational background, and current/ex-blue-collar worker). 

Being CWI was assessed with a question, “Do you take a drink consciously?”, and in conjunction with this question, the reason or aim for being CWI was asked. Therefore, being CWI implies trying to take a drink sufficiently in the present study.

The reasons for being CWI were asked to select from three options (multiple answers allowed); 1) prevention of cerebral stroke, 2) prevention of heat stroke, and 3) others in which individual reasons were described. For further analyses, we grouped the reasons into 4 criteria; “for prevention of heat stroke with/without the others excluding cerebral stroke”, “for prevention of cerebral stroke with/without the others excluding heat stroke”, “for prevention of both heat stroke and cerebral stroke with/without the others”, and “the others”. 

Height and body weight were measured with patients wearing socks and light clothing, and body mass index (BMI) was calculated by dividing weight in kilograms by the squared height in meters. The blood pressure was measured twice in each participant after a five-minute rest using an automatic sphygmomanometer (Nihon Colin, BP-103iⅡ), and the mean value for each participant was recorded. 

All blood samples were obtained in the morning after fasting for at least 10 hours, and blood samples were tested by one commissioned clinical laboratory center (SRL Inc., Tokyo, Japan). Blood glucose, blood urea nitrogen, serum sodium, serum potassium, and serum chloride were measured. Serum osmolarity (Osm/L) was calculated by Worthley’s formula: 2 × (serum sodium (mEq/L)) + (blood urea nitrogen (mg/dL))/2.8 + (glucose (mg/dL))/18 [29]. 

Daily salt intake was estimated using spot urine in the baseline survey according to the equation [30]; estimated daily salt intake = 21.98 × ((spot urine sodium (mEq/L)/spot urine creatinine (mg/dL)/10) × (PRCr))^0.392^/17. Predicted value of creatinine (PRCr) was calculated by the equation: PRCr = −2.04 × age + 14.89 × weight (kg) + 16.14 × height (cm) – 2244.45. 

### 2.4. Statistical Analysis

The participants were divided into two groups according to being CWI (CWI group) or not (non-CWI group). We evaluated the differences between the two groups. Student t-test was used for continuous variables and the chi-square test was used for categorical variables. We assessed 1) the association between being CWI and NAD and beverages intake, and 2) the association between the reasons for being CWI and NAD intake in the CWI group by using multivariate linear regression models after adjusting for sex, age, surveyed months (May–Oct. vs. Nov.–Apr.), BMI, hypertension (systolic blood pressure ≥ 140 mmHg, diastolic blood pressure ≥ 90mmHg, or taking antihypertensive), dyslipidemia (LDL ≥ 140 mg/dL, triglyceride ≥ 150mg/dL, or HDL < 40 mg/dL), current smoker, current drinker (excluded in analysis on alcohol intake), health conscious life habits, and socioeconomic factors; eating quickly, dinner within 2 hours before going to bed, late evening snack, skipping breakfast, eating until full stomach, regular use of vitamin tablets or supplements, walking habit (≥ 2 days/a week, for ≥ 30 minutes), daily use of a pedometer, sleeping time, good health status, living alone, educational background (bachelor or higher degree), current/ex-blue-collar worker, calculated serum osmolarity, and estimated daily salt intake. All significance tests were two-tailed, and *p* < 0.05 was considered significant in all analyses. All statistical analyses were performed with IBM SPSS Statistics for Windows version 22 (IBM Corp., Armonk, NY, USA).

### 2.5. Ethics Approval and Consent to Participate

This study was conducted according to the guidelines laid down in the Declaration of Helsinki and all procedures involving research study participants were approved by the Ethics Committees of the Institute of Biomedical Research and Innovation (committee approval number: 11–12) and Kyoto Koka Women’s University (committee approval number: 012). Written informed consent was obtained from all patients.

## 3. Results

### 3.1. Demographics Characteristics of the Participants and CWI

In 988 participants (698 women and 290 men, age 42–76), 644 (65.2%) participants replied that they are CWI and 344 (34.8%) participants replied they are not CWI (Table 1). There was no significant difference in sex between the CWI group and non-CWI group, although the proportion of women was relatively high in the CWI group. The mean age in the CWI group was significantly higher than that in the non-CWI group. The percentage of those who are CWI was significantly higher around summer (May–October) than around winter (November–April). The highest was 80.6% in September, and the lowest was 56.5% in February. As for healthy life habits, the proportion of walking habit and daily use of a pedometer were higher in the CWI group than in the non-CWI group; on the other hand, as for unhealthy life habits, dinner within 2 hours before going to bed was lower in the CWI group than in the non-CWI group. There was no significant difference in calculated serum osmolarity and estimated daily salt intake between the two groups. 

### 3.2. The Reasons for Being CWI According to Seasons

The reasons for being CWI are shown in Table 2. The most popular reason for being CWI was the prevention of heat stroke, followed by prevention of cerebral stroke. There was a difference in the popular reasons for being CWI according to the surveyed months; the most popular reason for being CWI around summer was for prevention of heat stroke (84.6% in Sep.), while it was for prevention of cerebral stroke around winter (43.1% in Jan.). After grouping these reasons into four criteria, “for prevention of heat stroke with/without the others excluding cerebral stroke” (n = 263), “for prevention of cerebral stroke with/without the others excluding heat stroke” (n = 104), “for prevention of both heat stroke and cerebral stroke with/without the others” (n = 84), and “the others” (n = 193), we found there was no association between the difference of the reasons for being CWI and the amount of the NAD intake. This finding was observed even after adjusting for age, sex, and surveyed months. 

### 3.3. The Amount of Beverage Intake and Its Details, According to CWI

The mean amount of NAD intake was 1717.4 ml/day, and sex difference was observed (women 1841.4 ± 672.9 ml/day vs. men 1485.3 ± 638.5 ml/day, *p* < 0.001). The difference in the amount of beverage between CWI and non-CWI groups was detailed in Table 3. The CWI group took significantly more NAD than the non-CWI group did. The number of those who drank less than 1000 ml NAD was observed to be 49 (7.6%) participants in the CWI group and 77 (22.4%) in the non-CWI group (*p* < 0.001). Total alcohol beverage intake (mL/day) in the CWI group was less than that in the non-CWI group, while there was no significant difference in its g/day of ethanol between the two groups. The CWI group drank decaffeinated beverages significantly more than the non-CWI group. There was no significant difference in the amount of total caffeine drink between them; however, green tea was taken more and coffee was taken less by the CWI group than by the non-CWI group. Surveyed months were associated with the amount of NAD intake, which was relatively high in the hot season; for example, the peak was seen in June (2048.5 ± 851.3 mL/day) and the bottom was seen in March (1514.8 ± 553.8 mL/day). This tendency was also observed after stratification of being CWI.

### 3.4. Association between Being Conscious of Water Intake and the Amount of Beverages Intake: Uni- and Multivariate Linear Regression Analyses.

The associations between CWI and the amount of NAD, alcohol, caffeine beverage, decaffeinated beverages, and total beverage intakes in multivariate regression models are presented in Table 4. Being CWI was positively associated with the amount of NAD intake after adjusting for sex, age, and surveyed months (model 1), and this association was observed even after adjusting for the other possible confounders (model 2). Being CWI was also positively associated with decaffeinated beverage intake in both models, but not with alcohol and caffeine beverage intakes. Similar findings were observed after stratification by sex. There was no significant association between the reasons for being CWI and NAD intake when linear regression analyses were conducted in CWI group.

## 4. Discussion

As far as we could investigate, this was the first study clarifying being CWI was positively associated with NAD, especially decaffeinated beverages, intake considering sex, age, surveyed months, BMI, hypertension, dyslipidemia, current smoker, current drinker, life conscious life habits, socioeconomic factors, and blood and urine samples in a healthy population. This association was not influenced by the reasons for being CWI. The popular reason for being CWI in winter was the prevention of cerebral stroke while that in summer was the prevention of heat stroke [14,31,32].

In general, drinking water is widely believed to be good for health [31,32]; therefore, it is reasonable that being CWI was positively associated with healthy life habits and negatively associated with unhealthy life habits in the present study. In spite of such a causal linkage between health awareness and being CWI, the present study demonstrated that being CWI was independently associated with sufficient NAD intake even after adjusting for these confounders in multivariate linear regression models. Therefore, being CWI is thought to be one of the independent healthy habits, and plays an important role in maintaining sufficient total water intake regardless of health awareness. 

Dehydration can cause various diseases; for example, heat stroke, vascular diseases, kidney stone, bladder cancer, colon cancer, and so on [14]. In accordance with that, the participants chose various reasons for being CWI, and heat stroke and cerebral stroke, especially in summer and in winter, respectively, were the most popular reasons for being CWI in the present study. While thinking of specific diseases is thought to be a potent motivation for taking NAD, the difference in the reasons for being CWI was not associated with the amount of NAD intake in the present study. Therefore, while the reasons for being CWI vary with the individual and it may be unnecessary to have any specific reason in mind in order to maintain sufficient NAD intake, cerebral stroke and heat stroke are simple examples implying the importance of taking water throughout the year.

It is unclear what kind of beverage is desirable or undesirable for maintaining sufficient water intake. For example, it is thought to be difficult to treat alcohol beverages and caffeine beverages, considering their diuretic effect [33]. As for alcohol, a recent study showed that alcoholic beverages contribute to moisture intake despite the diuretic effect of their ethanol content [34]. However, it goes without saying that taking lots of alcohol instead of water for health, or “for prevention of heat stroke” or “for prevention of cerebral stroke”, is generally considered to be nonsense. Therefore, it seems reasonable that the CWI group took less alcohol than the non-CWI group in the present study. On the other hand, as for caffeine, there would appear to be no clear basis for refraining from caffeine-containing drinks in situations where fluid balance might be compromised [35,36]. However, overuse of caffeine is known to be linked to potentially unhealthy behavior and unfavorable outcomes [37]. The knowledge of caffeine on health was not investigated in the present study, but the CWI group took much decaffeinated drink and refrained from coffee. They also took much green tea, but the concentration of caffeine in green tea is usually about 1/5 of the coffee. So, those who are CWI might place less significance on the caffeine of green tea. 

This study has some limitations. Firstly, the amount of food moisture was not measured, and water content was not calculated in each beverage. However, considering the overview that water intake is predominantly through consumption of drinking water and beverages (80%) plus water contained in food (20%) [14] and it is easy for individuals to control the amount of beverage intake, estimating daily NAD is thought to be useful for assessing water intake. Secondly, the amount of NAD intake might be inaccurate since it depended on self-assessment information. However, it is impractical for third-parties to confirm the volume of the beverages every time the participants drink. Therefore, in order to minimize errors, we standardized the interview method and conducted the face-to-face interview by medical professionals, such as research doctors, nurses, or nutritionists. Moreover, in the interviews, all answers to the questionnaires, which were distributed by post and were filled in beforehand, were reviewed; in particular, the size of cups or glasses used for each drink and the number of cups and glasses consumed daily were reviewed by reference to the examples of several sizes of cups. Thirdly, we did not assess the harmful effect of NAD intake in the present study. At least, however, nobody declared harmful effects, like water intoxication, at the interview and their blood test and urine test were normal in this respect while they had been taking NAD in a similar manner for a long time. However, nocturia has been reported to be one of risk factors of bone fracture in elderly people [38]. We should take it into consideration that the relatively healthy population in the present study might have influenced the findings. Fourthly, as for socioeconomic factors, the proportion of being CWI was lower in high educational group. Accordingly, univariate analyses showed high educational group and high occupational groups were inversely associated with NAD intake, although these were not significant in multivariate analyses in the present study. These findings might contradict the theory of socioeconomic status as a fundamental cause of diseases [39], but, considering a recent study which demonstrated an inverse occupational class gradient for stroke incidence in Japan in contrast to Western countries, this kind of socioeconomic factor may not be a universal phenomenon [40]. Finally, the amount of NAD intake in the present study recruiting a healthy population is not representative of the NAD intake everywhere, not only in the world but also in Japan [41], because NAD intake as well as water intake for healthy adults is thought to be generally influenced by external environment, such as climate, season, weather, temperature, and humidity [31,42]. At least, however, the finding that being CWI was positively associated with NAD intake should serve as a reference anywhere.

## 5. Conclusions

In conclusion, this cross-sectional study suggested being CWI independently played an important role in maintaining sufficient daily NAD intake regardless of its reasons and seasons; the popular reason was cerebral stroke in winter while it was heat stroke in summer. Practically, public education encouraging water intake is conducted around summer for prevention of heat stroke [43,44]; however, disruption of hydration status can occur in any season [15] and cerebral stroke also occurs in any season [19,20]. Therefore, not only in summer but also in any season, keeping hydration status by maintaining sufficient water intake is thought to be important to prevent ischemic stroke in all seasons.

## Figures and Tables

**Table 1:** Characteristics Demographic by consciousness of water intake.

	Overall (n = 988)	Consciousness of Water Intake		
		Conscious	Not Conscious	*p* Value
		(n = 644)	(n = 344)	
Women	698 (70.6%)	464 (72.0%)	234 (68.0%)	0.185
Age (y.o.)	61.0 ± 8.6	62.1 ± 8.1	58.9 ± 9.0	< 0.001
Height (m)	159.1 ± 7.7	158.6 ± 7.6	160.0 ± 7.8	0.009
Weight (kg)	54.8 ± 9.9	54.3 ± 9.5	55.5 ± 10.6	0.075
BMI(Kg/m^2^)	21.5 ± 2.8	21.5 ± 2.7	21.5 ± 3.0	0.707
Hypertension	124 (12.6%)	85 (13.2%)	39 (11.3%)	0.400
Dyslipidemia	459 (46.5%)	305 (47.4%)	154 (44.8%)	0.436
Surveyed months; May-Oct vs. Nov-Apr				< 0.001
May-Oct	434 (43.9%)	310 (48.1%)	124 (36.0%)	
Nov-Apr	554 (56.3%)	334 (51.9%)	220 (64.0%)	
Lifestyles				
Current Smoker	41 (4.1%)	21 (3.3%)	20 (5.8%)	0.055
Current Drinker	472 (47.8%)	297 (46.1%)	175 (50.9%)	0.154
Eating quickly (yes)	332 (33.6%)	209 (32.5%)	123 (35.8%)	0.295
Dinner within 2 hours before going to bed: ≥ 3 days/a week	138 (13.9%)	76 (11.8%)	62 (18.0%)	0.007
Late evening snack: ≥ 3 days/a week	191 (19.3%)	113 (17.5%)	78 (22.7%)	0.052
Skipping breakfast: ≥ 3 days/a week	58 (5.9%)	36 (5.6%)	22 (6.4%)	0.608
Eating until full stomach (yes)	588 (59.5%)	373 (57.9%)	215 (62.5%)	0.162
Regular use of vitamin tablets or supplements: once or more a week	350 (35.4%)	241 (37.4%)	109 (31.7%)	0.073
Walking habit: ≥ 2 days/a week, for ≥ 30 min.	557 (56.4%)	392 (60.9%)	165 (48.0%)	<0.001
Daily use of a pedometer	330 (33.4%)	240 (37.3%)	90 (26.2%)	<0.001
Sleeping time (hours)	6.1 ± 1.0	6.2 ± 0.9	6.1 ± 1.0	0.23
Good health status	837 (84.7%)	556 (86.3%)	281 (81.7%)	0.053
Socioeconomic factors				
Living alone	101 (10.2%)	66 (10.2%)	35 (10.2%)	0.971
Educational background (bachelor’s or higher degree)	355 (35.9%)	210 (32.6%)	145 (42.2%)	0.003
Current/ex- blue-collar worker	545 (55.2%)	355 (55.1%)	190 (55.2%)	0.974
Laboratory data				
Serum sodium (mEq/L)	143.4 ± 1.5	143.4 ± 1.5	143.3 ± 1.6	0.159
Serum potassium (mEq/L)	4.2 ± 0.3	4.2 ± 0.3	4.3 ± 0.3	0.475
Serum chloride (mEq/L)	104.2 ± 1.8	104.2 ± 1.8	104.1 ± 1.8	0.216
Blood urea nitrogen (mg/dL)	14.2 ± 3.2	14.4 ± 3.2	14.0 ± 3.3	0.122
Fasting blood glucose (mg/dL)	89.0 ± 8.0	89.2 ± 7.9	89.7 ± 8.2	0.316
Calculated serum osmolarity (Osm/L)	296.8 ± 3.5	297.0 ± 3.4	296.6 ± 3.6	0.058
Estimated daily salt intake (g/day)	8.4± 1.9	8.5 ± 1.8	8.3 ± 1.9	0.154

Continuous data was analyzed using student’s t test, and is shown in the mean ± standard deviation. Categorical data was analyzed using the χ2 test, and is shown as number (%). NAD; non-alcohol drink, BMI; body mass index, hypertension; systolic blood pressure ≥ 140mmHg, diastolic blood pressure ≥ 90mmHg or taking antihypertensive. Good health status; average, good and excellent among 5 grades (excellent, good, average, fair, bad). Calculated serum osmolarity = 2×(serum sodium) + (blood urea nitrogen)/2.8 + (glucose)/18

**Table 2:** The reasons for being conscious of water intake, according to seasons.

	Total (n = 644)		May-Oct. (n = 310)		Nov.-Apr. (n = 334)	
Prevention of heat stroke with/without other reasons	263	(40.8%)	163	(52.5%)	89	(26.6%)
Prevention of cerebral stroke with/without other reasons	104	(16.1%)	33	(10.6%)	71	(21.2%)
Prevention of both heat stroke and cerebral stroke with/without other reasons	84	(13.0%)	38	(12.2%)	46	(13.7%)
Limited to other reasons excluding prevention of heat stroke or cerebral stroke	193	(29.9%)	67	(21.6%)	131	(39.2%)
Free comments for being CWI other than heat stroke and cerebral stroke						
Prevention of dehydration	35	(5.4%)	10	(3.2%)	25	(7.4%)
Health	34	(5.2%)	10	(3.2%)	24	(7.2%)
Prevention of constipation	28	(4.3%)	12	(3.8%)	16	(4.7%)
Habit since young	27	(4.1%)	11	(3.5%)	16	(4.7%)
Sweating	21	(3.2%)	9	(2.9%)	12	(3.5%)
Thirst	20	(3.1%)	7	(2.2%)	13	(3.8%)
Preference for drinking NAD	10	(1.5%)	4	(1.2%)	6	(1.7%)
Prevention of renal stone	11	(1.7%)	3	(0.9%)	8	(2.3%)
Ameliorating blood viscosity	11	(1.7%)	3	(0.9%)	8	(2.3%)
Metabolism	9	(1.3%)	5	(1.6%)	4	(1.1%)
Maintaining renal function	9	(1.3%)	3	(0.9%)	6	(1.7%)
Prevention of gout	5	(0.7%)	1	(0.3%)	4	(1.1%)
Others	22	(3.4%)	8	(2.5%)	14	(4.1%)
Simple tabulation of free comments	242		86		156	

Multiple answers were allowed. NAD; non-alcohol drink.

**Table 3:** The amount of beverage intake and its details, according to consciousness of water intake.

	Overall (n = 988)	Conscious of Water Intake		
		Conscious	Not Conscious	*p* Value
		(n = 644)	(n = 344)	
Beverage intake				
Total beverage (ml/day)	1851.1 ± 704.2	1963.0 ± 693.6	1641.7 ± 676.5	< 0.001
Details of total beverage				
Total NAD (ml/day)	1717.4 ± 682.3	1841.4 ± 672.9	1485.3 ± 638.5	< 0.001
Total alcohol beverage (ml/day)	133.7 ± 242.1	121.6 ± 227.8	156.4 ± 265.8	0.040
Alcohol/total beverage	0.069 ± 0.114	0.058 ± 0.101	0.088 ± 0.134	< 0.001
Alcohol consumption (g/day of ethanol)	9.2 ± 17.6	8.5 ± 17.6	10.5 ± 17.6	0.086
Details of NAD intake				
Caffeine drink (ml/day)	1015.8 ± 611.5	1039.7 ± 612.1	971.2 ± 608.8	0.093
Coffee (n = 715)	428.6 ± 282.8	406.8 ± 268.1 (n = 457)	467.2 ± 303.8 (n=258)	0.008
Green tea (n = 721)	750.3 ± 489.7	781.8 ± 480.0 (n = 478)	688.2 ± 503.4 (n = 243)	0.015
Black tea (n = 216)	342.6 ± 257.0	333.0 ± 238.8 (n = 142)	361.0 ± 289.6 (n = 74)	0.448
Others with caffeine (n =124)	663.4 ± 472.9	688.6 ± 447.1 (n= 91)	593.7 ± 539.0 (n = 33)	0.325
Decaffeinated beverages (ml/day)	701.3 ± 587.9	801.7 ± 607.2	513.5 ± 498.9	< 0.001
Water (n = 576)	629.1 ± 418.6	678.6 ± 439.4 (n = 419)	497.2 ± 323.0 (n = 157)	< 0.001
Barley tea (n = 166)	815.6 ± 522.6	851.5 ± 500.5 (n = 107)	750.5 ± 559.0 (n = 59)	0.235
Milk or milk beverage (n = 404)	241.6 ± 132.0	243.9 ± 128.7 (n = 286)	236.1 ± 140.2 (n = 118)	0.593
Isotonic drink (n = 74)	385.8 ± 199.2	400.3 ± 192.6 (n = 59)	329.0 ±221.3 (n = 15)	0.267
Soy milk (n = 45)	227.1 ± 153.4	217.1 ± 160.9 (n = 32)	251.5 ± 135.8 (n = 13)	0.502
Others without caffeine (n = 213)	276.6 ± 213.6	281.3 ± 220.2 (n = 144)	267.0 ± 200.4 (n = 69)	0.649
Decaffeinated beverages/NAD	0.396 ± 0.274	0.426 ± 0.273	0.339 ± 0.269	< 0.001

Continuous data was analyzed using student’s t test, and is shown in the mean ± S.D. NAD; non-alcohol drink. For calculation of the mean ± S.D in each beverage of NAD, only the data of the participants who drink each beverage were used.

**Table 4:** Association between being conscious of water intake and the amount of beverages intake: Uni- and multivariate linear regression analyses.

Conscious of Water Intake	Univariate		Beta	*p* Value	Model 1		Beta	*p* Value	Model 2		Beta	*p* Value
	B	95% CI			B	95% CI			B	95% CI		
NAD intake												
Not conscious	ref.				ref.				ref.			
Conscious	356.1	(269.5, 442.8)	0.25	< 0.001	317.2	(230.7, 403.7)	0.22	< 0.001	318.1	(231.3, 405.0)	0.22	<0.001
Alcohol intake												
Not conscious	ref.				ref.				ref.			
Conscious	−34.8	(−55.4, −3.1)	-0.07	0.031	−21.3	(-51.2, 8.5)	−0.04	0.162	−22.5	(−48.1, 3.0)	-0.04	0.084
Caffeine beverage intake												
Not conscious	ref.				ref.				ref.			
Conscious	68.5	(−11.5, 148.6)	0.05	0.093	54.4	(−26.9, 135.7)	0.04	0.189	61.5	(−20.2, 143.3)	0.05	0.140
Decaffeinated beverage intake												
Not conscious	ref.				ref.				ref.			
Conscious	287.5	(212.6, 362.5)	0.23	< 0.001	262.8	(189.1, 336.5)	0.21	< 0.001	262.8	(189.1, 336.5)	0.21	< 0.001
Total beverage intake												
Not conscious	ref.				ref.				ref.			
Conscious	321.3	(231.2, 411.4)	0.22	< 0.001	295.9	(205.4, 386.3)	0.20	< 0.001	295.6	(205.4, 385.8)	0.20	< 0.001
NAD intake												
Not conscious	ref.				ref.				ref.			
Conscious	361.0	(253.2, 468.8)	0.24	< 0.001	334	(226.0, 442.1)	0.22	< 0.001	339.8	(230.5, 449.1)	0.23	< 0.001
Alcohol intake												
Not conscious	ref.				ref.				ref.			
Conscious	−31.4	(−58.0, −4.8)	−0.09	0.021	−25.3	(−52.4, 1.7)	0.07	0.066	−20.9	(−43.2, 1.4)	−0.06	0.066
Caffeine beverage intake												
Not conscious	ref.				ref.				ref.			
Conscious	92.8	(−5.9, 191.7)	0.07	0.065	76.4	(−24.1, 176.9)	0.06	0.136	83.3	(−18.6, 185.3)	0.06	0.109
Decaffeinated beverage intake												
Not conscious	ref.				ref.				ref.			
Conscious	268.1	(178.8, 357.4)	0.22	< 0.001	257.6	(170.1, 345.1)	0.21	< 0.001	256.5	(168.3, 344.7)	0.21	< 0.001
Total beverage intake												
Not conscious	ref.				ref.				ref.			
Conscious	329.6	(219.5, 439.7)	0.22	< 0.001	308.7	(198.2, 419.2)	0.20	< 0.001	318.9	(207.7, 430.2)	0.21	< 0.001
NAD intake												
Not conscious	ref.				ref.				ref.			
Conscious	324.6	(184.4, 464.7)	0.26	< 0.001	275.5	(134.2, 416.7)	0.22	< 0.001	310.6	(161.5, 459.7)	0.25	< 0.001
Alcohol intake												
Not conscious	ref.				ref.				ref.			
Conscious	−15.6	(−91.6, 60.0)	−0.02	0.684	−15.7	(−94.3, 62.8)	−0.02	0.694	−52.4	(−121.0, 16.0)	−0.08	0.133
Caffeine beverage intake												
Not conscious	ref.				ref.				ref.			
Conscious	−7.2	(−139.2, 124.7)	−0.01	0.914	0.67	(−136.3, 137.6)	0.00	0.992	14.3	(−129.4, 158.0)	0.01	0.845
Decaffeinated beverage intake												
Not conscious	ref.				ref.				ref.			
Conscious	331.8	(192.6, 471.1)	0.27	< 0.001	274.8	(135.7, 413.9)	0.22	< 0.001	296.3	(152.3, 440.2)	0.24	< 0.001
Total beverage intake												
Not conscious	ref.				ref.				ref.			
Conscious	308.9	(151.8, 466.0)	0.22	< 0.001	259.7	(101.6, 417.9)	0.19	0.001	258.1	(95.8, 420.3)	0.19	0.002

Model 1: adjusting for sex, age, and surveyed months; Model 2: model 1 plus adjusting for body mass index, hypertension (systolic blood pressure ≥ 140mmHg, diastolic blood pressure ≥ 90, or taking antihypertensives), dyslipidemia (LDL ≥ 140mg/dL, triglyceride ≥ 150mg/dL or HDL < 40mg/dL), current smoker, current drinker (excluded in analysis on alcohol intake), eating quickly, dinner within 2 hours before going to bed, late evening snack, skipping breakfast, eating until full stomach, regular use of vitamin tablets or supplements, walking habit (≥ 2 days/a week, for ≥ 30 minutes), daily use of a pedometer, sleeping time, good health status, living alone, educational background (bachelors or higher degree), current/ex- blue-collar worker, serum osmolality, and estimated daily salt intake. B; Non-standardized coefficients, Beta; Standardized coefficients.
